# What Personal and Environmental Factors Determine Frequency of Urban Greenspace Use?

**DOI:** 10.3390/ijerph110807977

**Published:** 2014-08-07

**Authors:** Martin Dallimer, Zoe G. Davies, Katherine N. Irvine, Lorraine Maltby, Philip H. Warren, Kevin J. Gaston, Paul R. Armsworth

**Affiliations:** 1 Sustainability Research Institute, School of Earth and Environment, University of Leeds, Leeds LS2 9JT, UK; 2 Durrell Institute of Conservation and Ecology (DICE), School of Anthropology and Conservation, University of Kent, Canterbury CT2 7NR, UK; E-Mail: z.g.davies@kent.ac.uk; 3 Social, Economic and Geographical Sciences Research Group, James Hutton Institute, Craigiebuckler, Aberdeen AB15 8QH, UK; E-Mail: Katherine.Irvine@hutton.ac.uk; 4 Department of Animal and Plant Sciences, University of Sheffield, Sheffield S10 2TN, UK; E-Mails: l.maltby@sheffield.ac.uk (L.M.); p.warren@sheffield.ac.uk (P.H.W.); 5 Environment and Sustainability Institute, University of Exeter, Penryn, Cornwall TR10 9FE, UK; E-Mail: k.j.gaston@exeter.ac.uk; 6 Ecology and Evolutionary Biology, University of Tennessee, Knoxville, TN 37996, USA; E-Mail: p.armsworth@utk.edu

**Keywords:** ecosystem services, psychological well-being, urban ecology, urbanisation, motivation

## Abstract

For many people, urban greenspaces are the only places where they encounter the natural world. This is concerning as there is growing evidence demonstrating that human well-being is enhanced by exposure to nature. There is, therefore, a compelling argument to increase how frequently people use urban greenspaces. This may be achieved in two complementary ways by encouraging: (I) non-users to start visiting urban greenspaces; (II) existing users to visit more often. Here we examine the factors that influence frequency of greenspace visitation in the city of Sheffield, England. We demonstrate that people who visit a site least frequently state lower self-reported psychological well-being. We hypothesised that a combination of socio-demographic characteristics of the participants, and the biophysical attributes of the greenspaces that they were visiting, would be important in influencing visit frequency. However, socio-demographic characteristics (income, age, gender) were not found to be predictors. In contrast, some biophysical attributes of greenspaces were significantly related to use frequency. Frequent use was more likely when the time taken to reach a greenspace was shorter and for sites with a higher index of greenspace neglect, but were unrelated to tree cover or bird species richness. We related these results to the motivations that people provide for their visits. Infrequent users were more likely to state motivations associated with the quality of the space, while frequent users gave motivations pertaining to physical, repeated activities. This suggests that there may be no simple way to manage greenspaces to maximise their use across user cohorts as the motivations for visits are very different.

## 1. Introduction

Over 50% of the world’s human population now lives in towns and cities [1]. For a substantial proportion of humanity, interactions with the natural world largely take place within an urban, human-dominated system. Urban greenspaces have a disproportionately important role in improving the quality of life of city dwellers. Amongst other properties (see [2,3,4] for comprehensive reviews), greenspaces have been shown to enhance physical and mental health, as well as other aspects of well-being [5,6,7,8,9,10,11]. There is, therefore, a compelling argument to increase the frequency with which people use urban greenspaces as a component of programs to improve public health and well-being.

The values and preferences people have for urban greenspaces have been linked to: (I) features of the spaces themselves, such as proximity/accessibility (e.g., [12]), safety/cleanliness (e.g., [13]), naturalness [14,15,16]; and, (II) the socio-demographic characteristics of individuals (e.g., [17]), suggesting that the benefits of greenspace use may not accrue equally to all sectors of society (*cf.* [18]). Equally, although the relationship between the natural environment in greenspaces (e.g., number of species, proportion of tree cover) and human well-being is not straightforward [19], it is possible that it may enhance the benefits experienced by visitors (e.g., [7,8,20]).

Public policy has increasingly acknowledged the need for high quality accessible urban greenspaces in order to encourage their recreational use [21,22]. There are two possible, complementary, approaches that could be followed to achieve this aim. First, policy could focus on promoting visits to greenspaces by those who are presently non-users. Second, existing infrequent users could be encouraged to make use of greenspaces more often. Here we examine the latter using a sub-set of data gathered as part of an interdisciplinary mixed-methods research programme aimed at understanding the importance of biodiversity in urban riparian greenspaces for city residents; results of other aspects of the study have been reported elsewhere [7,23,24].

We carry out our research in England, a region where approximately 90% of the population lives in towns and cities. Here, as in other heavily urbanised nations, there is a particularly pressing need to understand how the benefits that may be derived from use of urban greenspaces may be more widely experienced across the entire population. We ask the following three research questions:

(I) *Do frequent users of urban greenspaces report higher psychological well-being gains associated with their visit than less frequent users*? If there are health benefits which can be derived from visiting urban greenspaces, then we might expect people who are more regular users will experience greater benefits. We test this hypothesis using three measures of self-reported psychological well-being (reflection, attachment, continuity with past—see Section 2.2).

(II) *What determines how often people visit greenspaces*? If visiting greenspaces more frequently offers higher psychological well-being benefits, then we need to understand what drivers may be influencing frequent use. We hypothesise that this would be due to the biophysical characteristics [10,11,12,13,14,25] of the greenspaces, as well as the socio-demographic characteristics of the individuals concerned (e.g., [17]). We also explore the possibility that knowledge about wildlife could influence how often an individual might use a greenspace [7].

(III) *Are the motivations for visiting greenspaces different between high and low frequency users*? People can express many different reasons for using urban greenspaces, which are not limited to the prosaic, such as proximity or convenience (e.g., [9,26]). We therefore hypothesise that frequent visitors may well express different reasons for their visits than those who go to greenspaces less often.

**Figure 1:**
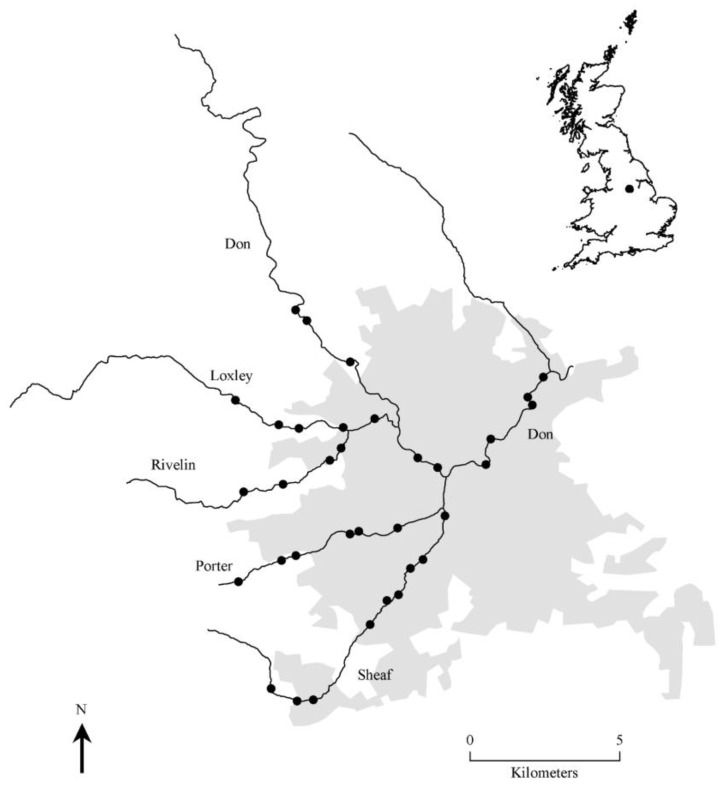
The urban area of Sheffield, United Kingdom (shaded), showing the major rivers running through the city (solid lines) and study sites (filled circles). The inset shows the location of Sheffield in Britain.

## 2. Methods

### 2.1. Study System

The study was conducted in Sheffield (53°22′N, 1°20′W), the fifth largest city in England with a human population of 520,700. As Sheffield lies at the confluence of several rivers, riparian areas offer an important recreational resource for its residents, especially as the rivers are distributed throughout the city in areas that are urban and suburban, as well as the more rural periphery. Thirty-four sites with public access were selected to represent the range of riparian greenspaces available to city dwellers, spanning a wide geographic area across all rivers (Figure 1), many of which are publicly owned/managed and semi-natural in character (e.g., woodland, brownfield sites, open locations).

### 2.2. Characterising Visitors to Greenspaces

To address our three research questions we drew upon open- and closed-ended questions embedded within a longer structured survey assessing values associated with biodiversity and the natural world [7,24]. All study materials underwent ethics review and verbal consent was obtained from participants following a brief description of the study. We wished to engage with as wide a range of people using the riparian zones as possible. Therefore, each site was visited at least four times, covering daytime and early evening during weekends and weekdays, using a rule of thumb of approaching every third person who passed by the study site. Over half (54.3%) of those asked to participate did so, yielding 1108 completed questionnaires (median = 34 per site). Participants were predominantly of European ethnicity (91.7%; broadly in line with Sheffield’s population as a whole, which is 91.2% of European descent), represented both genders (62% male), and covered a broad array of age groups (16 to 70+) and household income (below £10,000 to above £70,000 per year). We acknowledge that our sample is self-selected (*i.e.*, we only interview those people who have already chosen to visit greenspaces). However, our intention is not to understand what differentiates users from non-users, but to quantify the drivers of usage frequency among existing users.

For research question (I) (*Do frequent users of urban greenspaces report higher psychological well-being gains associated with their visit than less frequent users*?), visit frequency was quantified through a closed-ended question which asked how often an individual came to that particular riparian greenspace. Possible responses were daily, weekly, monthly and less than monthly. We used self-reported psychological well-being measures to assess the benefits of greenspace usage, in line with previous research [7,8] and theoretical considerations including reflection/contemplation [14,25,27] and sense of place (e.g., [28,29]). Seven statements measured reflection/contemplation, while a further 14 assessed sense of place constructs. Participants answered on a 5-point Likert scale (1 = strongly disagree, 5 = strongly agree) in response to the stem question “Please indicate how much you agree with each statement about this stretch of river and the neighbouring banks”. We then used factor analysis (principal axis factoring; oblique rotation) to identify meaningful interpretable well-being factors: reflection (ability to think and gain perspective), attachment (degree of emotional ties with the stretch of river), and continuity with past (extent to which sense-of-identity is linked to the stretch of river through continuity across time). Continuous measures were derived by calculating the participant’s average rating of the set of statements forming each factor (see [7] for full details of the measures) (Table 1).

To address question (II) (*what determines how often people visit greenspaces*?) our questionnaire included closed-ended socio-demographic questions. In addition, we assessed participants’ knowledge about the natural world via a wildlife identification skill test [7]. This was done by asking people to identify photographs of four species of bird, butterfly and plant commonly encountered within our sites. We used the answers to generate continuous measures of “wildlife knowledge” by summing the number of correct responses to give a score from 0 to 12 for each participant (Table 1). We also assessed the biophysical properties of our study sites, as outlined in Section 2.3 below.

**Table 1:** For 1108 visitors to 34 riparian greenspaces in Sheffield, England, site-level medians (range) for: biophysical site properties, self-reported psychological well-being of visitors (measured on a 1–5 scale: 1 = strongly disagree to 5 = strongly agree, in response to the stem question “*Please indicate how much you agree with each statement about this stretch of river and the neighbouring banks*”) and the socio-demographic characteristics of visitors. Wildlife identification skill gives the median number of correctly identified images (up to a maximum of 12 in total). Household income before tax is given in GBP thousands *per annum*.

Variable	Median (Min–Max)
Biophysical site properties	
Travel time (minutes)	10 (1–340)
Number of bird species	12 (4–18)
Tree cover (proportion)	0.37 (0.05–0.91)
Greenspace neglect	2 (0–6)
Psychological well-being	
Reflection	3.99 (3.26–4.43)
Attachment	4.32 (3.42–4.67)
Continuity with past	3.26 (2.40–3.86)
Wilidlife Knowledge	
Wildlife identification skill (number of photographs out of 12 correctly identified)	2.09 (0.78–3.17)
Socio-demographic characteristics	
Gender	62% male
Age	40 (18–70)
Household income	£20 (£10–£75)
Study sample size	
Number of participants	34 (10–46)

For question (III) (*are the motivations for visiting greenspaces different between high and low frequency users*?), we elicited peoples’ own descriptions of why they were visiting a particular greenspace. To do this, we included an open-ended question (“As for today, what are the two main reasons that brought you to this stretch of river?”) [9]. To minimise the potential influence of subsequent closed-ended questions on responses, this question was asked first; seven individuals did not provide an answer. Responses from the remaining 1,101 participants were iteratively content-analysed [30] by two researchers (KNI/MD) following the rationale, analysis protocol and identified taxonomy from Irvine *et al.* [9] as a guide. Visit-motivation responses were first sorted into codes (e.g., the comments “countryside in a very urban setting” and “see some nature” were both placed in a “Natural Setting” code) informed by participants’ language; Kappa analysis [31] indicated a substantial agreement between the two researchers who were independently assigning codes (90.4%; Kappa = 0.89). All mismatched codes were resolved by consensus agreement. Following the general approach of content analysis [30], codes were then grouped into descriptive themes, the development of which was informed by commonly mentioned words/phrases and meanings within the codes, previous research findings and theoretical constructs. Themes were subsequently categorised into nine domains grounded in existing theoretical constructs regarding the relationship between people and nature [14,32,33], holistic models of health [33] and previous research (e.g., [9,34]). Here we concentrate on the domain and theme level data only.

### 2.3. Characterising the Biophysical Properties of the Study Greenspaces

Urban river corridors show a high degree of environmental variation and can support diverse biological communities (e.g., [35]). In Sheffield, previous work has shown that there is substantial variation in the biophysical properties of these riparian greenspaces [23,36], which could influence the decisions people make regarding how often to visit. For the purposes of this study we characterized each study site using four different metrics (Table 1): (I) site accessibility; (II) number of bird species; (III) proportion of tree cover; and (IV) neglect/maintenance. We deliberately excluded the presence/absence of built facilities, such as a café or playground, as a possible explanatory variable for visit frequency as these were present on only three sites.

Site accessibility was quantified by using the proxy of travel time, which was measured by asking each participant to state how long (in minutes) it took them to reach the greenspace. We included the number of bird species as birds play a central role in human-wildlife interactions in England [37]: (I) bird feeding is a common and widespread activity [38,39]; (II) bird watching is a popular and fast growing leisure interest [40]; (III) bird-focussed citizen science initiatives successfully engage large numbers of people [41,42]; and, (IV) birds are more likely to be recognised by the general public than other common and widespread plant and animal groups [7]. The number of bird species was surveyed on each of the 34 sites. Following standard protocols [43], two visits were made in spring and early summer to coincide with the breeding season, with the second at least six weeks after the first. To ensure that the maximum number of species was encountered, visits began between one and three hours after sunrise (the time of highest avian activity) and were only carried out in suitable weather conditions (low wind, no rain or mist). A single observer (MD) recorded the identity of each bird that was seen or heard from the survey point (the same location at which questionnaires were administered) over a five minute period (see [23] for details of bird and ecological survey methods and complete results). A list of all species encountered during both visits was collated (Table 1), among them only three species (feral geese, feral pigeon *Columbia livia* and the rose-ringed parakeet *Psittacula krameri*) were non-native, with the latter species only observed on a single occasion [7,23].

Tree cover is a further highly visible aspect of the natural world, readily appreciated and noted by visitors. Tree cover was mapped in a Geographical Information System (GIS) by manually tracing around each tree or group of trees shown in aerial photographs [44]. The proportion of cover in a 50 m radius around each location was then determined. Finally, we included a metric of greenspace neglect. This was derived from field surveys where we counted the number of large (e.g., furniture) and small (e.g., food packaging) items of litter that were on both banks and in the river channel itself in an area 40 m up and downstream from the location where participants were invited to complete the questionnaire. For both large and small items, each site was given a score of 0 where no litter was present, 1 where the amount of litter was less than the average across all sites, or 2 where the amount of litter was greater than average. Sites were also characterised by the presence of graffiti and abandoned buildings (both scored 1/0 for presence/absence). All four scores were combined into a single greenspace neglect index which had a theoretical maximum of 6 and minimum of 0 (Table 1).

### 2.4. Statistical Analyses

We undertook the following statistical analyses to answer our research questions:

(I) *Do frequent users of urban greenspaces report higher psychological well-being gains associated with their visit than less frequent users?* We used Kruskal-Wallis tests to determine whether there were significant differences in self-reported well-being between visit-frequency categories.

(II) *What determines how often people use greenspaces*? Using an ordered logistic regression approach, we modelled visit frequency as the response variable against a suite of explanatory variables including site biophysical properties (travel time, number of bird species, tree cover, greenspace neglect), the socio-demographic make-up of participants (age, gender, income) and their ability to identify wildlife. Ordered logistic regression is an extension of logistic regression (used when the response is binary) that can be employed when there are more than two possible responses and where the order of responses is informative. Ordered logistic regression assumes that the parameter estimates that describe the relationship between the highest category of the response variable and all other categories are the same as those that describe the relationship between the next highest category and all others, hence there is a single parameter estimate for each variable. All analyses were carried out using the “polr” command in the MASS package [45] of the R statistical software [46]. The model coefficients can be difficult to interpret due to the log scale, so we converted them into proportional odds ratios by taking the exponential of the coefficients. The outputs can then be understood in the same way as odds ratios from a standard logistic regression model.

(III) *Are the motivations for visiting greenspaces different between high and low frequency users*?

We used chi-squared tests to determine whether the frequency of the types of motivation given at the domain and theme levels varied according to visit frequency.

## 3. Results

(I) *Do frequent users of urban greenspaces report higher psychological well-being gains associated with their visit than less frequent users*? All three axes of self-reported psychological well-being varied significantly according to visit frequency (Kruskal-Wallis tests: reflection: χ^2^ = 9.323, df = 3, *p* = 0.025; attachment: χ^2^ = 9.388, df = 3, *p* = 0.0246; continuity with past: χ^2^ = 30.571, df = 3, *p* < 0.001). In all cases well-being was lowest for those individuals who visited greenspaces least often. However, it was only for the continuity with past well-being axis, that those individuals visiting most frequently reported the highest scores (Table 2).

**Table 2:** For 1108 visitors to 34 riparian greenspaces in Sheffield, England, median (interquartile range) self-reported psychological well-being for three axes, according to the frequency with which participants visit the study greenspace.

	Visit Frequency			
Well-being axis	Daily	Weekly	Monthly	Less than monthly
Reflection	4.00 (3.58–4.43)	4.00 (3.57–4.29)	4.00 (3.57–4.43)	3.86 (3.43–4.29)
Attachment	4.33 (3.83–4.83)	4.33 (3.83–5.00)	4.50 (4.00–5.00)	4.17 (3.83–5.00)
Continuity with past	3.40 (2.80–4.0)	3.20 (2.60–3.80)	3.20 (2.40–3.80)	3.00 (2.40–3.40)

(II) *What determines how often people visit greenspaces*? The socio-demographic characteristics (household income, age, gender) and wildlife identification skill of our participants were not significant predictors of more frequent use of greenspaces (Table 3). In contrast, some biophysical properties of greenspaces were significantly related to visit frequency. Participants were more likely to be frequent users if their travel time to a site was lower. Visit frequency was, however, not determined by the number of bird species present, nor the amount of tree cover. Counter-intuitively, participants were more likely to state they used a site frequently where the greenspace neglect index was high (Table 3).

(III) *Are the motivations for visiting greenspaces different between high and low frequency users*? Content analysis of participants’ comments identified nine motivation domains (Table 4 and Table 5). The majority of responses fell within two domains (Physical and Space Qualities), which broadly relate to reasons pertaining to the physical body or associated with physically tangible and less prosaic, intangible characteristics of the greenspace itself respectively. 

**Table 3:** Ordered logistic regression exploring the relationship between visit frequency, the biophysical properties of the visited greenspace, the socio-demographic characteristics of the respondent and their ability to identify wildlife.

Explanatory Variable	Parameter ^1^	*T* Value	*p* Value	Odds Ratio
Travel time	−0.015 (0.002) ^2^	−6.642	0.000	0.985 (0.980–0.989)
Number of bird species	0.031 (0.023)	1.320	0.187	1.031 (0.985–1.079)
Tree cover	−0.059 (0.292)	−0.203	0.839	0.942 (0.531–1.672)
Greenspace neglect	0.148 (0.043) ^2^	3.425	0.001	1.160 (1.066–1.263)
Wildlife identification skill	−0.053 (0.029)	−1.829	0.067	0.948 (0.896–1.004)
Household income	−0.002 (0.003)	−0.616	0.538	0.998 (0.991–1.004)
Age	−0.004 (0.004)	−0.898	0.369	0.996 (0.988–1.004)
Gender (Male) ^3^	0.111 (0.124)	0.892	0.372	1.117 (0.876–1.426)

^1^ Parameter estimates (standard errors) are in units of ordered logs and can be difficult to interpret. Odds ratios (95% CI) also presented, calculated by taking the exponential of the parameter estimate. Interpretation as follows: for a one unit increase in the greenspace neglect index score, the odds of moving from “less than monthly” to “monthly” (or upwards from any category to the next most frequent category) are multiplied by 1.160; ^2^ Statistically significant explanatory; ^3^ coded as a dummy. Odds ratio here represents the odds of moving from one category to the next higher category if the respondent was male, rather than female.

Prior to further analysis, all remaining domains were merged into a single “Other” category. At the domain level, less frequent visitors were more likely to give Space Quality motivations and frequent visitors Physical motivations (χ^2^ = 36.058, N = 1977, df = 6, *p* < 0.001) (Table 6).

**Table 4:** From a total of 1977 responses to an open-ended question “*As for today, what are the two main reasons that brought you to this stretch of river*?”, provided by visitors to riparian greenspaces in Sheffield, England, visit motivations were coded into domains. For analytical purposes all domains other than Space Qualities and Physical were placed into a single Other category.

Domain	Responses
Space Qualities	604
Physical	1156
Affective	7
Children	31
Cognitive	57
Global	1
Social	39
Spiritual	6
Unstructured Time	69

**Table 5:** From a total of 1977 responses to an open-ended question “*As for today, what are the two main reasons that brought you to this stretch of river*?”, provided by visitors to riparian greenspaces in Sheffield, England, visit motivations were coded into domains (Table 4);. Of the 1745 responses from the domains Space Qualities and Physical, the number coded into each theme

Domain	Theme	Responses
Space Qualities	Nature	246
	Park Features	285
	“Sense of Place” ^1^	68
Physical	Physical Pursuits	1085
	Physical Restoration	61

^1^ Due to the low number of comments within the place attachment and place identity themes—which highlighted the less prosaic/intangible qualities of the greenspace as reasons for visiting—a single amalgamated theme was used in analysis.

**Table 6:** Number of responses to an open-ended question “*As for today, what are the two main reasons that brought you to this stretch of river*?” provided by visitors to greenspaces in Sheffield, England, the number of motivations falling into different domains according to visit frequency.

	Domain		
Visit frequency	Other	Physical	Space Qualities
Daily	40	305	159
Weekly	55	412	189
Monthly	32	168	82
Less than monthly	88	259	169

Within the two major domains, five themes were identified (Table 5). At the theme level there were again significant differences in the motivations stated by participants who visit the sites at different levels of frequency. Nature-related motivations (from the Space Quality domain; Table 5) were more often given by people who were infrequent users of the greenspace (χ^2^ = 13.988, N = 599, df = 3, *p* < 0.01). In contrast, Physical Pursuit (χ^2^ = 106.68, df = 3, N = 1146, *p* < 0.01) and Park Features (which include both physically tangible and less prosaic intangible) motivations (χ^2^ = 24.211, df = 3, N = 599, *p* < 0.01) were more likely to be provided by those visiting sites more often (Table 7).

**Table 7:** Number of responses to an open-ended question “*As for today, what are the two main reasons that brought you to this stretch of river*?” provided by visitors to greenspaces in Sheffield, England, the number of motivations falling into different themes according to visit frequency.

	Themes in the Space Qualities Domain			Themes in the Physical Domain	
Visit frequency	Nature	Park Features	“Sense of Place”	Physical Pursuits	Physical Restoration
Daily	47	100	12	294	14
Weekly	48	102	11	397	19
Monthly	77	27	8	158	17
Less than monthly	77	57	33	253	11

## 4. Discussion

As more people’s lives are dominated by urban experiences, the gap between humans and the natural world is growing [47]. With the degree of urbanisation increasing, there is mounting concern about the effects both on biodiversity [48] and human health/well-being [49]. In response, an urban greening movement has been promoted by researchers (e.g., [50]) and policy-makers alike (e.g., [21,22]), keen to capitalise on the benefits to humans of urban greenspace provision.

Here, we find that the least frequent users of greenspaces state the lowest health benefit (in terms of self-reported psychological well-being). For one metric (continuity with past), very frequent, daily use corresponds with individuals stating the highest well-being gains. This result highlights once again the importance of outdoor recreation and the provision of greenspace, which can improve human health and well-being (e.g., [3,11]). Demonstrating a link between qualities of the natural environment, such as number of bird species or the amount of tree cover with facets of human well-being could deliver substantive benefits for urban biodiversity conservation. If this link is to be fully exploited, it is essential we understand what ecological components of urban greenspaces are associated with increased frequency of use by those who visit them. However, we uncovered no evidence to suggest that enhancing the number of bird species or proportion of tree cover in a greenspace would result in a rise in the frequency with which existing users choose to visit the site. Our previous work has shown that self-reported psychological well-being of greenspace users in Sheffield is positively related to the number of bird species on a site [7], raising hopes that, for certain taxonomic groups, there may be an opportunity to align urban biodiversity conservation with the urban greening human health agenda. Nonetheless, our findings here show no clear link between visit frequency and greenspace ecological condition (as measured by the number of bird species or tree cover). As such it may well be challenging for conservation biologists to mesh their priorities with other reasons for enhancing greenspaces [51,52].

We did not find that the socio-demographic characteristics of participants were significant drivers of greenspace use frequency. This is in contrast to previous studies which have shown that nature-based recreational activities (*i.e.*, those not restricted to urban greenspaces) are unequally distributed across society. The less affluent tend to make fewer, less frequent trips to natural environments (e.g., [17]). Similarly, residents of more deprived areas make up a disproportionately small number of those found using protected areas for recreation in northern England [53]. Comparable patterns have been shown in South Africa [54] and the US [55]. Furthermore, we found no relationship between knowledge of the natural world (here assessed via a measure of wildlife identification skill) and the frequency with which participants visited a particular greenspace. Although using a very different metric (ours is a metric of knowledge of the natural world), this contrasts with Lin *et al.* [26], whose data indicated that householders reporting a higher connectedness to natural world were more likely to have visited an urban greenspace in the previous week. Instead, we find that the frequency with which participants visit urban greenspaces is determined by how long it takes an individual to reach a site.

Our index of site neglect/maintenance (the greenspace neglect indicator) was, somewhat counter-intuitively, positively related to visit frequency. There are a number of possible explanations for this result. First, it may be that greenspaces that are closer to where people live are more heavily littered and graffitied. Similarly, it may be the case that sites contain more litter because they are visited more frequently (*i.e.*, the visitor pressure results in higher levels of litter than would otherwise be found).

By asking participants to state the motivations for their visit, we were able to explore the lack of a direct relationship between some aspects of the natural environment (number of bird species and tree cover), socio-demographic characteristics, wildlife knowledge and visit frequency. We found significant differences in the motivations that people gave depending on how frequently they visited the greenspace. Frequent visitors were more likely to state motivations situated in the Physical domain and Physical Pursuits theme. Motivations from the Space Qualities domain and Nature theme were more prevalent in less frequent visitors. This implies that there may be no obvious way of moving people from the infrequent to the frequent user category, as the underlying motivations for visits are different.

## 5. Conclusions

Allying biodiversity conservation to public health as part of the urban greening agenda depends on a better understanding of the interactions between people and nature [2,56]. We were able to demonstrate a generally positive association between how often people use a greenspace and self-reported well-being. Moreover, we show that accessibility (in the form of the time taken to reach a greenspace) is an important factor in determining how frequently existing users visit them. However, we find no clear link between visit frequency and greenspace ecological condition (as measured by number of bird species and tree cover). Priority should therefore be given to improving accessibility and availability of greenspaces by providing more green infrastructure close to where residents live or work. Such an approach would offer many additional benefits for both people and biodiversity, not least because the proportion of an urban area covered by green infrastructure has been strongly linked to enhancing a range of ecosystem services at the city scale [57].

There are a number of ways our work could be extended. First, there are several additional characteristics of an individual that may influence the frequency with which they use an urban greenspace; self-reported mood and personal attitudes towards the natural world (e.g., [26]) are two that warrant further investigation. Second, our study was carried out *in situ* and asked about use of a specific site. A parallel survey at the household level may reveal different relationships between usage of greenspaces more generally and socio-demographic characteristics. Finally, we were solely interested in urban greenspace usage. Many people may also visit the countryside for recreation purposes and this may have an impact on their usage of and motivations for use, of urban greenspaces.
